# Correspondence

**Published:** 1870-11

**Authors:** E. Holderness

**Affiliations:** Towanda, McLean Co., Ill.


### Correspondence.

TOWANDA, MCLEAN CO., ILL., *Oct. 15, 1870.*

DR. N. S. DAVIS,—DEAR SIR,—Herein I send you an imperfect report of a case that occurred in my practice a few days since—the first of the kind that has occurred to me in a practice of over twelve years.

On the night of the 11th inst., I was called upon to visit a child some five months old, that was reported to be suffering intensely from a colic, and had passed some blood. I visited the child as soon as I could—it being some miles in the country—and found that the child had been suffering excruciating pain, coming on every few minutes, since about 4 o'clock the previous evening—it now being about 3 o'clock in the morning. At the time of my arrival the child was not suffering as much as it had been. Thinking that the trouble, whatever it was, might be subsiding, I gave the child one drop of tinct. Opii., and directed that it be repeated as often as every four hours, if the child still continued to suffer, and at the same time to use warm water injections, with a few drops of oil turpentine in each injection. The parents stated that the child had always been troubled with costiveness. After staying some two or three hours I left, with the injunction that if the child should get worse again, to let me know immediately. About 12 o'clock of the same day I was sent for again, saying that the child was no better, and had been passing more blood. At this time it occurred to me that it must be a concealed *hernia*, or an *intussusception*, and that I must prepare myself accordingly.

I found the child about the same as it had been the previous night, and at short intervals straining to stool, but passing nothing but blood, and as no fecal matter had passed, I felt satisfied that there must be an obstruction in the bowels some place.

I again examined the child for hernia, but finding none, concluded that it must be an invaginated bowel, and upon passing my finger—previously well oiled—up the rectum, I found the bowel hanging down within itself, and tried to replace it, but it would return as my finger was withdrawn. As the case was still recent, I thought best to reduce the displaced bowel if possible. I had no other means at hand than a valvular India Rubber ball syringe and warm water. I managed to inject near one-half gallon of water into the child's bowels, which satisfied me that I had found a hole somewhere, and upon examination, to my infinite satisfaction, found the Intussusception gone.

I directed the continued use of the opeate as before, to keep the bowels quiet for a time, and bowels to be filled with warm water again if the trouble should return.

At this date the child is apparently well.

Yours respectfully,

E. HOLDERNESS.

